# Current Status of Pancreatic Exocrine Insufficiency Diagnosis and Pancreatic Enzyme Replacement Therapy Implementation in Patients With Chronic Pancreatitis

**DOI:** 10.1002/jgh3.70473

**Published:** 2026-09-29

**Authors:** Noriharu Itoh, Yuko Hasebe, Toru Yada, Kazutaka Nozawa

**Affiliations:** ^1^ Medical Affairs, Viatris Pharmaceuticals Japan G.K. Minato‐ku Tokyo Japan; ^2^ EPS Corporation Shinjuku‐ku Tokyo Japan

**Keywords:** chronic pancreatitis, exocrine pancreatic insufficiency, insurance claims analysis, nutritional status, nutritional support

## Abstract

**Aims:**

The prevalence of chronic pancreatitis (CP) is increasing in Japan. This study aimed to elucidate the current status of pancreatic exocrine insufficiency (PEI) diagnosis and the prescription of pancreatic enzyme replacement therapy (PERT) in Japanese patients with CP.

**Methods:**

We conducted a retrospective observational study using a large‐scale claims database in Japan with a data collection period between April 2008 and August 2022. Patients with CP were categorized into PERT and no‐PERT cohorts based on the presence or absence of PERT prescriptions.

**Results:**

We found an increase in the number of CP diagnoses, PERT prescriptions, and PEI diagnoses over the past decade. The median PERT persistence time was 254.0 days (95% confidence interval: 219.0–288.0), and the 5‐year persistence rate was 20.4%. Patients in the PERT cohort (*n* = 3056) were younger (61.9 vs. 65.4 years), had a higher mean Charlson Comorbidity Index (1.7 vs. 1.3), and were more likely to be underweight (body mass index < 18.5 kg/m^2^, 13.7% vs. 6.0%) than those in the no‐PERT cohort (*n* = 32 861). Patients in the PERT cohort had higher rates of in‐hospital deaths (6.3% vs. 4.7%), weight loss (13.3% vs. 9.2%), pancreatic diabetes (1.7% vs. 0.7%), and pancreatic cancer (1.7% vs. 0.7%) than those in the no‐PERT cohort.

**Conclusion:**

The number of PEI diagnoses and PERT prescriptions for patients with CP has increased in Japan over the past decade. The low PERT persistence rate suggests the need for further research to understand the factors that affect treatment persistence in CP management.

## 1 Introduction

Chronic pancreatitis (CP) is a multifactorial pathological fibroinflammatory syndrome of the pancreas characterized by the progressive loss of endocrine and exocrine functions [1]. In addition to common risk factors, such as alcohol consumption and tobacco use, genetic predisposition, chronic obstructive causes, metabolic disorders, and autoimmune disorders can contribute to CP development [1]. As the disease progresses, patients with CP experience abdominal pain [2], diabetes mellitus caused by endocrine insufficiency, maldigestion or malabsorption caused by exocrine insufficiency, and an increased risk of pancreatic cancer (PC) [1, 3]. Nationwide surveys in Japan have shown that the prevalence of CP increased from 28.5 per 100 000 persons in 1994 [4] to 44.5 per 100 000 persons in 2016 [5].

The prevalence of pancreatic exocrine insufficiency (PEI) increases with age in apparently healthy individuals [6, 7], and PEI is observed in > 30% of patients with CP [8, 9]. A 1‐week, double‐blind, randomized, placebo‐controlled study found that pancreatic enzyme replacement therapy (PERT) improved fat and nitrogen absorption and reduced stool fat, stool frequency, and stool weight compared to the placebo in patients with CP and PEI [10]. A subsequent 6‐month open‐label extension study found that PERT was well tolerated and resulted in significant weight gain from baseline [11]. Additionally, a prospective multicenter questionnaire study found that dietary counseling and PERT prescriptions improved symptoms, including steatorrhea and pain, and enhanced quality of life, in patients with CP [12]. Although a fat‐restricted diet has often been used to treat patients with abdominal pain in the compensated stage of CP, the Japanese clinical practice guidelines for the decompensated stage of CP with PEI state that patients should receive PERT and adequate nutrition without fat restriction [13].

Pancrelipase, a drug containing high‐titer pancreatic enzymes (including amylase, lipase, and protease), was launched in Japan in August 2011 for treating PEI caused by the decompensated stage of CP, pancreatectomy, cystic fibrosis, and other conditions. This launch was based on the results of phase 3 studies, which showed a significant improvement in fat absorption with pancrelipase compared to a placebo and a long‐term beneficial impact on nutritional endpoints [14]. The clinical practice guidelines for chronic pancreatitis published by the Japanese Society of Gastroenterology recommend the use of pancrelipase in patients with PEI and associated steatorrhea and weight loss [15]. However, the real‐world usage and effectiveness of PERT have not been well studied. Moreover, the Japanese clinical diagnostic criteria for CP were revised in 2019 [16] and have increased the specificity for early‐stage CP diagnosis compared with the previous version, which may have affected the number of patients treated with PERT.

Therefore, we conducted a retrospective observational study using a large‐scale claims database with the aim of investigating the current status of PEI diagnosis and PERT prescription in Japanese patients with CP. In this study, we assessed the real‐world use and persistence of PERT. We also explored the factors associated with the outcomes of patients with CP.

## 2 Methods

### 2.1 Study Design

This retrospective observational cohort study used the Medical Data Vision (MDV) database (Medical Data Vision Co. Ltd., Tokyo, Japan). MDV is a health claims database comprising anonymized data from all insurance types in inpatient and outpatient settings at 28% of advanced care hospitals using the Diagnosis Procedure Combination (DPC) system in Japan as of December 2023 [17, 18]. A unique identification number was assigned to each patient in each DPC hospital.

The data were collected from April 2008 to August 2022 (Supporting Information Digital Content Figure S1). The inclusion period (April 2009 to August 2022) was divided into the following terms: Term 1, between April 2009 and July 2011 (before the launch of pancrelipase); Term 2, between August 2011 and December 2018 (after the launch of pancrelipase, before the CP diagnostic criteria change in 2019); and Term 3, between January 2019 and August 2022 (after the CP diagnostic criteria change in 2019). The PERT start date was defined as the first date of the prescription for pancrelipase (LipaCreon Granules or LepaCreon Capsules, Viatris Pharmaceuticals Japan G.K., Minato‐ku, Tokyo, Japan). The index date was the first date of CP diagnosis (prevalent CP cohort, overall incident CP cohort, and incident CP cohort [no PERT]) or the start date of PERT after the first date of CP diagnosis (incident CP cohort [PERT]). The follow‐up period was from the index date to either the earliest date of in‐hospital death, date of the last observation, or end of the study period.

This study was conducted in accordance with the legal, regulatory, and ethical requirements. Based on the applicable legal requirements in Japan, approval from the institutional review board and informed consent from patients were not required for this study, as it used anonymized data.

### 2.2 Patient Selection

Patients were selected from the MDV database using the International Statistical Classification of Diseases and Related Health Problems 10th Revision (ICD‐10) codes. Inclusion in the prevalent CP cohort (Supporting Information Digital Content Figure S1A) was based on the following criteria: (i) registration in the MDV database between April 2009 and August 2022 and (ii) a confirmed diagnosis of CP (ICD‐10 codes K86.0, K86.1, or K85.9, and disease names associated with CP, excluding suspected cases). Patients were excluded from the prevalent CP cohort if they had a confirmed diagnosis of malignancy with ICD‐10 codes of C00‐C97 (excluding suspected cases) within 1 year after the first CP diagnosis date.

Patients in the prevalent CP cohort were included in the incident CP cohort (Supporting Information Digital Content Figure S1B) if they met the following criteria: (i) their database start date was at least 1 year before the first confirmed CP diagnosis, and (ii) data were available in the database within 1 year before the first confirmed CP diagnosis. Patients were excluded from the incident CP cohort if they received PERT prescriptions the month before the first confirmed CP diagnosis.

Patients in the incident CP cohort were included in the PERT cohort (Supporting Information Digital Content Figure S1C) if they met the following criteria: (i) they had PERT prescriptions after the first confirmed CP diagnosis, (ii) their PERT start date was after the launch of pancrelipase (August 2011), and (iii) they had data within 1 year before the PERT start date. Patients in the incident CP cohort were included in the no‐PERT cohort if they did not receive PERT prescriptions after their first confirmed CP diagnosis.

### 2.3 Data Collection and Outcomes

Information on patient characteristics, CP treatment, and the duration of PERT implementation (based on the first and last prescription date and the day's supply) was collected. Nutritional status was assessed using the Controlling Nutritional Status (CONUT) score, modified Glasgow prognostic score (mGPS), and neutrophil‐to‐lymphocyte ratio (NLR). Information on the following outcome events was collected: in‐hospital death, weight loss (> 4.5 kg or > 5% loss per year), comorbidities (pancreatic diabetes, osteoporosis, cachexia, fracture, PC > 1 year after the index date, and malignancy > 1 year after the index date [including PC]). PEI was identified based on a PEI‐related disease with an ICD‐10 code of K86.8, diagnosed by treating physicians, rather than on pancreatic function or steatorrhea‐related test results.

### 2.4 Statistical Analysis

Descriptive statistics were used to summarize continuous variables as medians (ranges) or means (standard deviations) and categorical variables as counts (%). For exploratory purposes, the Kaplan–Meier method was used to estimate the median time to outcome events and discontinuation of PERT after the index date, with a 95% confidence interval (CI). The median event‐free survival time was measured as days from the index date to the first occurrence of any of the aforementioned outcome events. Missing data were not imputed except for the diagnosis date, which was defined as the first day of the month in medical claims. For exploratory purposes, univariate and multivariate analyses were performed using the Cox proportional hazards regression model to assess the association between the outcome events and covariates. The stepwise method was used to select covariates with 0.05 as the alpha‐to‐enter and alpha‐to‐remove significance levels for the following covariates: sex, age, CP type, Charlson Comorbidity Index (CCI), comorbidities, steatorrhea‐related test, treatment, and concomitant drugs. All analyses were performed using SAS software (version 9.4, SAS Institute, Cary, NC, USA).

## 3 Results

### 3.1 Patient Characteristics

Among the 158 659 patients who were diagnosed with CP between April 2009 and August 2022, 103 064 patients were included in the prevalent CP cohort, and 35 925 patients in the incident CP cohort (Figure 1). Patients in the incident CP cohort were divided into PERT (*n* = 3056) and no‐PERT cohorts (*n* = 32 861) based on the presence or absence of PERT prescriptions.

**FIGURE 1:**
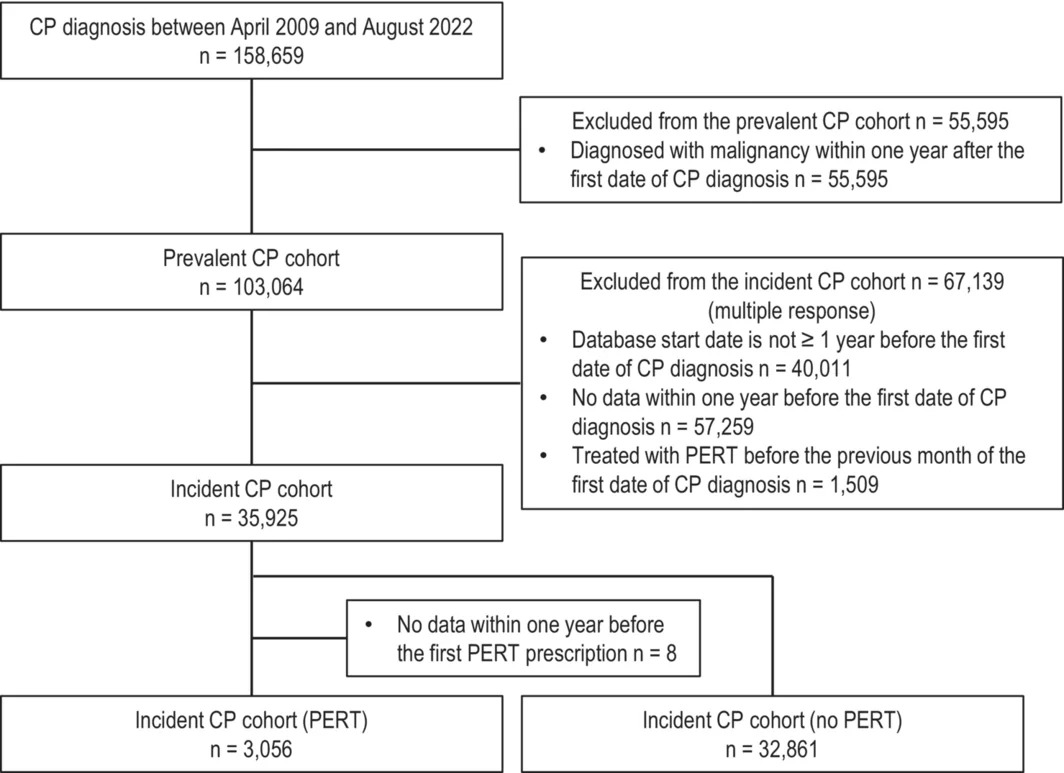
Patient flowchart. CP, chronic pancreatitis; PERT, pancreatic enzyme replacement therapy.

Compared with patients in the no‐PERT cohort, those in the PERT cohort were younger (61.9 years vs. 65.4 years) and had a higher mean CCI (1.7 vs. 1.3), and the proportions of smokers (29.9% vs. 15.4%), patients who were underweight (body mass index [BMI] < 18.5 kg/m^2^, 13.7% vs. 6.0%), and patients with the CP type of “alcoholic chronic pancreatitis” (10.0% vs. 5.5%) or “chronic pancreatitis acute exacerbation” (17.7% vs. 8.2%) were higher in this cohort (Table 1). The proportion of patients with comorbidities (e.g., PEI, diabetes mellitus, and abdominal pain) diagnosed within 1 year before the index date was higher in the PERT cohort than in the no‐PERT cohort. In the PERT cohort, the proportion of patients diagnosed with PEI within 1 year before the index date increased from 29.4% (410 of 1396) in Term 2 to 46.0% (763 of 1660) in Term 3.

**TABLE 1:** Patient characteristics.

	Prevalent CP cohort	Incident CP cohort		
		Overall	PERT	No PERT
	(*N* = 103 064)	(*N* = 35 925)	(*N* = 3056)	(*N* = 32 861)
Male, *n* (%)	63 346 (61.5)	21 985 (61.2)	1977 (64.7)	20 003 (60.9)
Age, years, mean (SD)	64.5 (15.6)	65.0 (15.9)	61.9 (16.0)	65.4 (15.8)
BMI, kg/m^2^	*n* = 16 532	*n* = 13 095	*n* = 1698	*n* = 11 844
Mean (SD)	22.14 (5.06)	22.22 (5.24)	21.20 (3.97)	22.30 (5.34)
< 18.5, *n* (%)	2957 (2.9)	2256 (6.3)	420 (13.7)	1970 (6.0)
CP type (multiple responses), *n* (%)				
Alcoholic chronic pancreatitis	6813 (6.6)	2178 (6.1)	307 (10.0)	1801 (5.5)
Chronic pancreatitis	97 866 (95.0)	34 079 (94.9)	2451 (80.2)	31 241 (95.1)
Chronic pancreatitis acute exacerbation	10 107 (9.8)	3414 (9.5)	540 (17.7)	2690 (8.2)
CCI^a^				
Mean (SD)	0.6 (1.3)	1.3 (1.7)	1.7 (1.9)	1.3 (1.7)
0, *n* (%)	77 460 (75.2)	15 824 (44.0)	930 (30.4)	14 402 (43.8)
1, *n* (%)	11 491 (11.1)	9099 (25.3)	874 (28.6)	8327 (25.3)
2, *n* (%)	6148 (6.0)	4741 (13.2)	484 (15.8)	4396 (13.4)
≥ 3, *n* (%)	7965 (7.7)	6261 (17.4)	768 (25.1)	5736 (17.5)
Smoking, *n* (%)				
No	8459 (8.2)	6637 (18.5)	638 (20.9)	6130 (18.7)
Yes	7197 (7.0)	5713 (15.9)	915 (29.9)	5062 (15.4)
Unknown	87 408 (84.8)	23 575 (65.6)	1503 (49.2)	21 669 (65.9)
Comorbidities^a^, *n* (%)				
PEI	608 (0.6)	24 (0.1)	1173 (38.4)^d^	14 (0.0)
Diabetes mellitus	8629 (8.4)	6873 (19.1)	850 (27.8)	6339 (19.3)
Osteoporosis	3428 (3.3)	2715 (7.6)	272 (8.9)	2521 (7.7)
Cachexia	1 (0.0)	1 (0.0)	0 (0.0)	1 (0.0)
Steatorrhea	8 (0.0)	2 (0.0)	20 (0.7)	1 (0.0)
Abdominal pain	2030 (2.0)	1649 (4.6)	367 (12.0)	1407 (4.3)
Abdominal distension	523 (0.5)	425 (1.2)	90 (2.9)	362 (1.1)
Pneumonia	702 (0.7)	566 (1.6)	56 (1.8)	520 (1.6)
Pancreatic stones	468 (0.5)	394 (1.1)	190 (6.2)	335 (1.0)
Fracture	2457 (2.4)	1919 (5.3)	170 (5.6)	1778 (5.4)
Weight loss	116 (0.1)	104 (0.3)	26 (0.9)	87 (0.3)
Treatment and tests^a^, *n* (%)				
Pancreatic function diagnostic test	98 (0.1)	70 (0.2)	104 (3.4)	44 (0.1)
Nutritional guidance	3905 (3.8)	3333 (9.3)	484 (15.8)	3033 (9.2)
Steatorrhea‐related tests				
Fecal lipids	6 (0.0)	3 (0.0)	2 (0.1)	2 (0.0)
Fecal smear	102 (0.1)	66 (0.2)	51 (1.7)	42 (0.1)
Concomitant drugs^a^, *n* (%)				
Camostat	5241 (5.1)	3878 (10.8)	738 (24.1)	3617 (11.0)
Nafamostat	2899 (2.8)	2316 (6.4)	356 (11.6)	2062 (6.3)
Trepipton	68 (0.1)	54 (0.2)	3 (0.1)	50 (0.2)
CONUT score^b^	*n* = 1413	*n* = 1284	*n* = 117	*n* = 1198
Mean (SD)	2.31 (2.35)	2.23 (2.27)	3.69 (3.39)	2.16 (2.20)
Normal (0–1), *n* (%)	656 (0.6)	605 (1.7)	40 (1.3)	577 (1.8)
Mild malnutrition (2–4), *n* (%)	554 (0.5)	510 (1.4)	40 (1.3)	475 (1.4)
Moderate malnutrition (5–8), *n* (%)	157 (0.2)	134 (0.4)	21 (0.7)	117 (0.4)
Severe malnutrition (9–12), *n* (%)	46 (0.0)	35 (0.1)	16 (0.5)	29 (0.1)
mGPS^c^, *n* (%)				
Normal (0)	1405 (1.4)	1240 (3.5)	120 (3.9)	1123 (3.4)
Undernutrition (0)	306 (0.3)	260 (0.7)	36 (1.2)	228 (0.7)
Precachexia (1)	375 (0.4)	324 (0.9)	31 (1.0)	293 (0.9)
Cachexia (2)	530 (0.5)	460 (1.3)	70 (2.3)	422 (1.3)
NLR	*n* = 2402	*n* = 2167	*n* = 184	*n* = 2017
Mean (SD)	0.52 (0.30)	0.52 (0.30)	0.50 (0.35)	0.52 (0.30)

Abbreviations: BMI, body mass index; CCI, Charlson comorbidity index; CONUT, controlling nutritional status; CP, chronic pancreatitis; CRP, C‐reactive protein; mGPS, modified Glasgow prognostic score; NLR, neutrophil‐to‐lymphocyte ratio; PEI, pancreatic exocrine insufficiency; PERT, pancreatic enzyme replacement therapy; SD, standard deviation.

^a^ Based on data within a year before the index date (the first date of CP diagnosis or the PERT start date after the first date of CP diagnosis).

^b^ The sum of albumin, lymphocyte, and total cholesterol scores.

^c^ Patients with available CRP and albumin levels were assessed. Normal (CRP < 0.5 mg/dL, albumin > 3.5 g/dL), undernutrition (CRP < 0.5 mg/dL, albumin ≤ 3.5 g/dL), precachexia (CRP ≥ 0.5 mg/dL, albumin > 3.5 g/dL), and cachexia (CRP ≥ 0.5 mg/dL, albumin ≤ 3.5 g/dL).

^d^ 29.4% (410/1396) in Term 2 (between August 2011 and December 2018) and 46.0% (763/1660) in Term 3 (between January 2019 and August 2022).

### 3.2 PERT Prescriptions and PEI Diagnoses

The number of patients with CP diagnoses, PERT prescriptions, and PEI diagnoses increased over time during the study period up to 2021 in the prevalent cohort and up to 2019 in the incident CP cohort (Figure 2A,B). The proportion of patients with PERT prescriptions among those with CP in the prevalent cohort increased from 3.4% in 2012 (1 year after the approval of pancrelipase) to 9.8% in 2021 (Figure 2A). In the prevalent cohort, there was a discrepancy between the number of patients with PERT prescriptions and those with PEI diagnoses, suggesting that not all patients who received PERT had a PEI diagnosis, and not all patients diagnosed with PEI received PERT. The proportion of patients with PERT prescriptions in the incident CP cohort was stable between 2012 and 2021 at approximately 7.4%–9.5% (Figure 2B). Similarly, the proportion of patients with PERT prescriptions increased from 8.1% in Term 2 to 12.3% in Term 3 in the prevalent CP cohort and remained stable at 8.2% in Term 2 and 8.6% in Term 3 in the incident CP cohort (Supporting Information Digital Content Table S1). In the incident CP cohort, the number of patients with PERT prescriptions was higher than the number of patients with PEI diagnoses, suggesting that patients diagnosed with PEI were highly likely to receive PERT. In the PERT cohort, the number of patients diagnosed with PEI increased until 2021 (Figure 2C), and the proportion of patients diagnosed with PEI increased from 47.3% in Term 2 to 54.5% in Term 3. In contrast, the proportion of patients with a PEI diagnosis in the no‐PERT cohort remained stable at 0.3% in Term 2 and 0.4% in Term 3.

**FIGURE 2:**
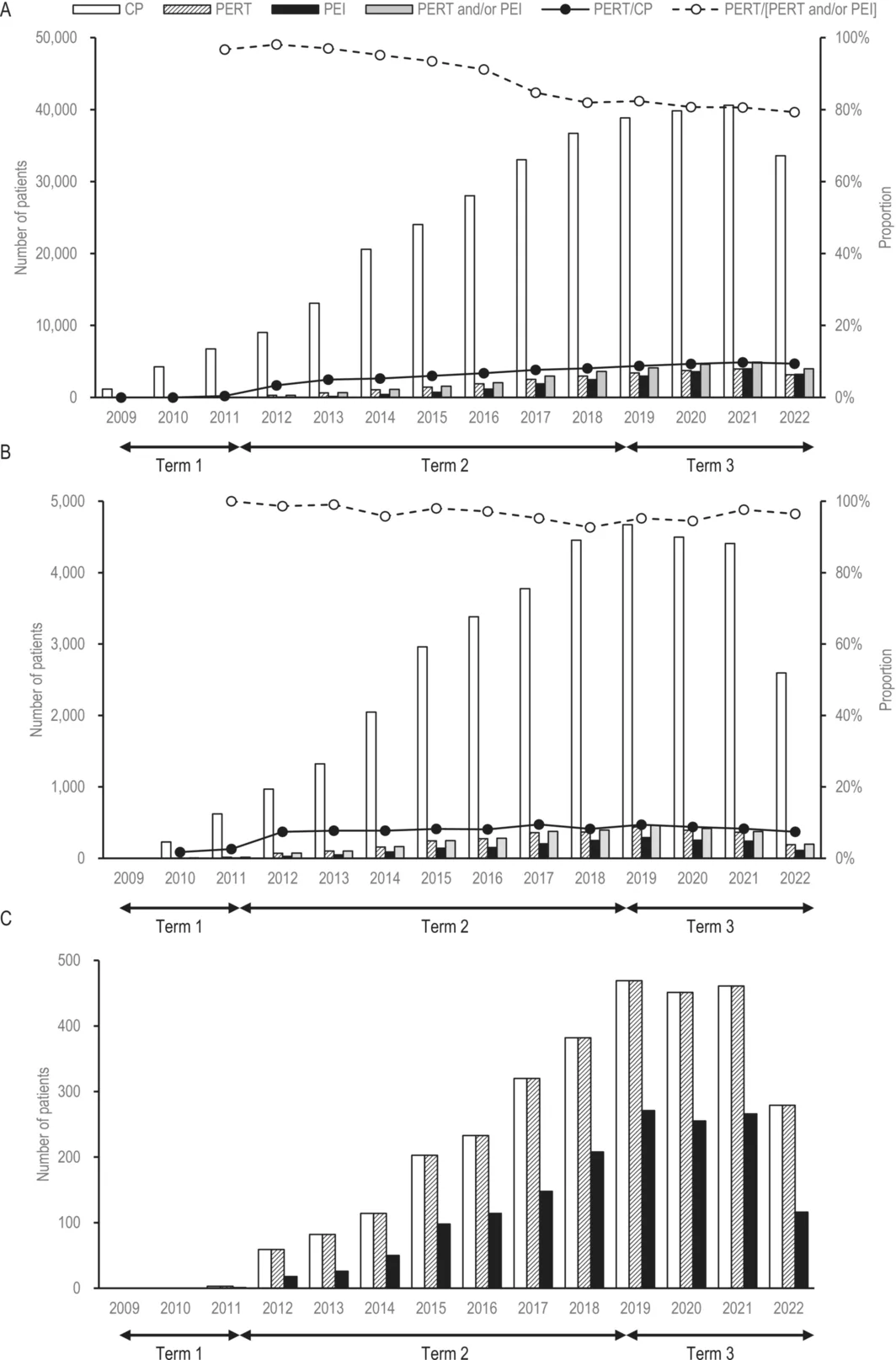
Number of patients with CP diagnoses (white bar), PERT prescriptions (diagonal bar), PEI diagnoses (black bar), and PERT and/or PEI (gray bar) in each year, with the proportion of PERT to CP (black circle and line) and PERT to PERT and/or PEI (white circle and broken line) in the prevalent CP cohort (A), incident CP cohort (B), and PERT cohort (C). Term 1 is between April 2009 and July 2011, Term 2 is between August 2011 and December 2018, and Term 3 is between January 2019 and August 2022. CP, chronic pancreatitis; PEI, pancreatic exocrine insufficiency; PERT, pancreatic enzyme replacement therapy.

In the incident CP cohort, the number of patients with PERT prescriptions decreased with each prescription (Figure 3). For any prescription, 900 and 1800 mg daily doses accounted for approximately 40% each, and a daily dose > 1800 mg accounted for less than 1%.

**FIGURE 3:**
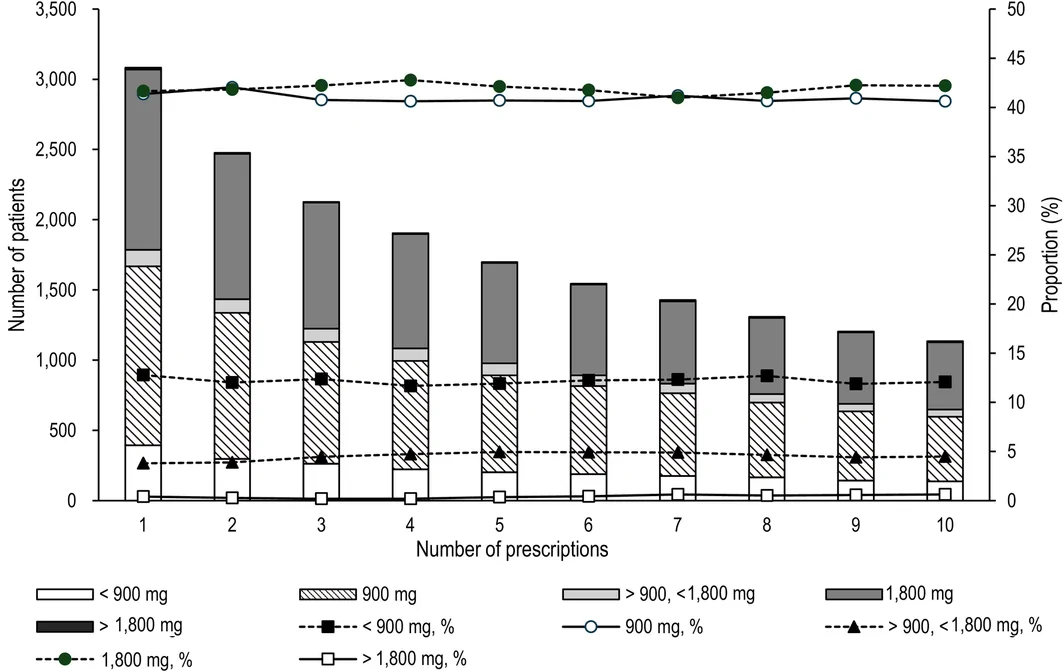
Number and proportion of patients who had each PERT (pancrelipase) dose at first to tenth prescriptions in the incident CP cohort. Patients prescribed PERT after the end of the follow‐up period were included. CP, chronic pancreatitis; PERT, pancreatic enzyme replacement therapy.

Around 82.9% (2534 of 3056) of patients in the incident CP (PERT) cohort started PERT within 1 year from the first date of CP diagnosis (Supplemental Digital Content Table S2), with 61.1% (1867 of 3056) starting within 1 month. The proportion of patients who started PERT within 1 year of CP diagnosis remained stable at 86.7% at Term 2 and 79.8% at Term 3.

### 3.3 PERT Persistence Rates Stratified by Patient Characteristics

The median PERT persistence time in the overall PERT cohort was 254.0 days (95% CI: 219.0–288.0, Table 2). The PERT discontinuation rate was 57.3% (1752 of 3056), with 1‐, 3‐, and 5‐year persistence rates of 44.3%, 28.1%, and 20.4%, respectively. When stratified by the time from CP diagnosis to the PERT start date, the median persistence time was shorter in patients who started PERT within 1 month of the first CP diagnosis than in those who started PERT within > 1 and ≤ 3 months or > 3 and ≤ 6 months. The median persistence time was longer when the CP type was “chronic pancreatitis acute exacerbation” than when it was “alcoholic chronic pancreatitis” or “chronic pancreatitis” and when PEI, diabetes mellitus, camostat, pancreatic function diagnostic test (PFD), or nutritional guidance were present versus absent.

**TABLE 2:** PERT persistence rates stratified by patient characteristics in the PERT cohort.

	*N*	Discontinuation, *n* (%)	1‐year persistence rate (95% CI), %	3‐year persistence rate (95% CI), %	5‐year persistence rate (95% CI), %	Median persistence time (95% CI), days
Overall	3056	1752 (57.3)	44.3 (42.2, 46.3)	28.1 (26.0, 30.2)	20.4 (18.1, 22.8)	254.0 (219.0, 288.0)
Time from the first date of CP diagnosis to the PERT start date						
≤ 1 month	1867	1105 (59.2)	39.2 (36.6, 41.7)	22.9 (20.4, 25.6)	17.7 (14.9, 20.7)	187.0 (155.0, 210.0)
> 1 and ≤ 3 months	326	164 (50.3)	54.7 (48.5, 60.5)	40.0 (33.3, 46.6)	29.8 (22.4, 37.7)	539.0 (334.0, 857.0)
> 3 and ≤ 6 months	153	80 (52.3)	55.1 (45.8, 63.5)	33.0 (23.3, 43.0)	27.9 (17.9, 38.8)	476.0 (317.0, 743.0)
> 6 month and ≤ 1 year	188	119 (63.3)	49.7 (41.8, 57.1)	30.6 (22.8, 38.6)	19.6 (12.0, 28.4)	364.0 (182.0, 525.0)
CP type						
Alcoholic chronic pancreatitis	307	191 (62.2)	42.2 (36.0, 48.2)	26.8 (20.8, 33.2)	20.4 (14.3, 27.4)	238.0 (187.0, 344.0)
Chronic pancreatitis	2451	1499 (61.2)	45.6 (43.5, 47.8)	29.1 (26.9, 31.4)	21.1 (18.7, 23.7)	287.0 (245.0, 326.0)
Chronic pancreatitis acute exacerbation	540	335 (62.0)	53.8 (49.3, 58.1)	33.6 (28.9, 38.3)	24.4 (19.4, 29.7)	440.0 (340.0, 503.0)
PEI						
No	1883	1100 (58.4)	41.9 (39.3, 44.4)	27.9 (25.3, 30.5)	21.1 (18.4, 24.0)	214.0 (188.0, 259.0)
Yes	1173	652 (55.6)	48.0 (44.7, 51.1)	27.6 (23.9, 31.3)	17.8 (13.7, 22.3)	336.0 (261.0, 401.0)
Diabetes mellitus						
No	2206	1306 (59.2)	42.4 (40.1, 44.8)	25.2 (22.8, 27.6)	18.7 (16.2, 21.4)	214.0 (196.0, 252.0)
Yes	850	446 (52.5)	48.8 (45.0, 52.5)	35.7 (31.5, 39.9)	24.9 (19.9, 30.2)	346.0 (301.0, 455.0)
Camostat						
No	2318	1341 (57.9)	41.3 (39.0, 43.6)	25.5 (23.1, 27.9)	19.1 (16.5, 21.9)	208.0 (182.0, 238.0)
Yes	738	411 (55.7)	53.0 (48.9, 56.9)	35.4 (31.1, 39.7)	24.2 (19.6, 29.1)	440.0 (336.0, 567.0)
PFD						
No	2952	1702 (57.7)	43.4 (41.3, 45.4)	27.1 (25.0, 29.3)	19.9 (17.6, 22.3)	238.0 (209.0, 272.0)
Yes	104	50 (48.1)	66.8 (56.0, 75.6)	52.4 (40.3, 63.2)	35.9 (21.9, 50.2)	1185.0 (610.0, 2104.0)
Nutritional guidance						
No	2572	1494 (58.1)	43.1 (40.9, 45.3)	26.7 (24.5, 29.0)	20.3 (17.9, 22.8)	230.0 (203.0, 268.0)
Yes	484	258 (53.3)	50.1 (45.1, 55.0)	34.9 (29.3, 40.5)	16.5 (9.0, 26.1)	377.0 (289.0, 527.0)

*Note:* Values are based on data at the index date (PERT start date) or within a year before the index date.

Abbreviations: CI, confidence interval; CP, chronic pancreatitis; PEI, pancreatic exocrine insufficiency; PERT, pancreatic enzyme replacement therapy; PFD, pancreatic function diagnostic test.

### 3.4 CP‐Related Outcome Events

Compared with patients in the no‐PERT cohort, those in the PERT cohort experienced more in‐hospital deaths (6.3% vs. 4.7%), weight loss (13.3% vs. 9.2%), pancreatic diabetes (1.7% vs. 0.7%), and PC (1.7% vs. 0.7%), and less osteoporosis (4.3% vs. 7.3%) and fractures (8.5% vs. 9.1%, Table 3). The median event‐free survival time was shorter in the PERT cohort than in the no‐PERT cohort (2386.0 vs. 3442.0 days).

**TABLE 3:** Outcomes during follow‐up in the incident CP cohort.

	Incident CP cohort	
	PERT	No PERT
	*N* = 3056	*N* = 32 861
Event (earliest), *n* (%)		
In‐hospital death	192 (6.3)	1552 (4.7)
Weight loss (> 4.5 kg or > 5% loss per year)	405 (13.3)	3018 (9.2)
Pancreatic diabetes	53 (1.7)	229 (0.7)
Osteoporosis	131 (4.3)	2411 (7.3)
Cachexia	0 (0.0)	2 (0.0)
Fracture	260 (8.5)	3001 (9.1)
Pancreatic cancer (> 1 year after the index date)	52 (1.7)	221 (0.7)
Malignancy^a^ (> 1 year after the index date)	144 (4.7)	1415 (4.3)
Median event‐free survival time (95% CI), days	2386.0 (1987.0, 3117.0)	3442.0 (3317.0, 3900.0)

*Note:* The follow‐up period started at the first date of CP diagnosis for “no PERT” and the PERT start date after the first date of CP diagnosis for “PERT.”

Abbreviations: CI, confidence interval; CP, chronic pancreatitis; PERT, pancreatic enzyme replacement therapy.

^a^ Including pancreatic cancer.

### 3.5 Covariates Associated With Outcome Events

In the incident CP cohort, univariable Cox regression analysis revealed that the risk of CP‐related outcome events was positively correlated with male sex, older age, lower weight, lower BMI, alcoholic CP, higher CCI, fecal smear implementation, diabetes mellitus, abdominal pain, pneumonia, fracture, nutritional guidance implementation, concomitant drugs (camostat and nafamostat), higher inflammation (C‐reactive protein [CRP]), higher CONUT score, lower NLR, and undernutrition and cachexia categories in the mGPS (Table 4). Multivariable analysis revealed that the risk of CP‐related outcomes was positively correlated with older age, alcoholic CP, higher CCI, fecal smear implementation, abdominal pain, fracture, nutritional guidance implementation, and concomitant drugs (camostat and nafamostat) and negatively correlated with osteoporosis and weight loss (Table 4).

**TABLE 4:** Covariates associated with outcome events in the incident CP cohort.

Variables	Reference	Univariable				Multivariable			
		*n*	Estimate	95% CI	*p*‐value	*n*	Estimate	95% CI	*p*‐value
Female	Male	35 925	−0.0771	−0.1219, −0.0324	0.0007				
Age, years	(continuous)	35 925	0.0220	0.0204, 0.0236	< 0.0001	35 925	0.0217	0.0201, 0.0234	< 0.0001
Weight, kg	(continuous)	13 442	−0.0068	−0.0090, −0.0046	< 0.0001				
BMI, kg/m^2^	(continuous)	13 095	−0.0088	−0.0161, −0.0015	0.0175				
CP type	Alcoholic CP	35 925	−0.2737	−0.3560, −0.1913	< 0.0001	35 925	−0.3801	−0.4638, −0.2965	< 0.0001
CCI	(continuous)	35 925	0.1158	0.1047, 0.1269	< 0.0001	35 925	0.0858	0.0741, 0.0975	< 0.0001
Smoking, No	Yes	12 350	0.0456	−0.0164, 0.1076	0.1493				
Steatorrhea‐related test (fecal smear), No	Yes	35 925	−0.5738	−0.9923, −0.1554	0.0072	35 925	−0.5812	−1.0002, −0.1623	0.0065
Comorbidities, No	Yes								
PEI		35 925	−0.0438	−0.9202, 0.8326	0.9220				
Diabetes mellitus		35 925	−0.2424	−0.2926, −0.1921	< 0.0001				
Osteoporosis		35 925	0.0488	−0.0285, 0.1261	0.2163	35 925	0.3698	0.2872, 0.4524	< 0.0001
Cachexia		35 925	5.0000	−69.6815, 79.6816	0.8956				
Steatorrhea		35 925	7.0002	−73.9400, 87.9403	0.8654				
Abdominal pain		35 925	−0.1575	−0.2555, −0.0596	0.0016	35 925	−0.2067	−0.3052, −0.1083	< 0.0001
Abdominal distension		35 925	−0.1452	−0.3254, 0.0350	0.1143				
Pneumonia		35 925	−0.2743	−0.4253, −0.1233	0.0004				
Pancreatic stones		35 925	0.0965	−0.1148, 0.3077	0.3708				
Fracture		35 925	−0.5670	−0.6469, −0.4870	< 0.0001	35 925	−0.4464	−0.5315, −0.3613	< 0.0001
Weight loss		35 925	0.4100	−0.0653, 0.8852	0.0909	35 925	0.5117	0.0356, 0.9878	0.0352
Treatment, No	Yes								
PFD		35 925	0.2260	−0.2644, 0.7164	0.3664				
Nutritional guidance		35 925	−0.4107	−0.4774, −0.3441	< 0.0001	35 925	−0.2403	−0.3095, −0.1711	< 0.0001
Concomitant drugs, No	Yes								
Camostat		35 925	−0.3486	−0.4111, −0.2861	< 0.0001	35 925	−0.1872	−0.2526, −0.1218	< 0.0001
Nafamostat		35 925	−0.4779	−0.5531, −0.4028	< 0.0001	35 925	−0.4134	−0.4921, −0.3346	< 0.0001
Trepipton		35 925	−0.4560	−0.9185, 0.0065	0.0533				
CRP	(continuous)	2569	0.0324	0.0151, 0.0496	0.0002				
CONUT score	(continuous)	1284	0.1823	0.1425, 0.2221	< 0.0001				
mGPS, Undernutrition (0)	Normal (0)	1500	1.0426	0.8285, 1.2568	< 0.0001				
mGPS, Precachexia (1)	Normal (0)	1564	0.2077	−0.0425, 0.4579	0.1038				
mGPS, Cachexia (2)	Normal (0)	1700	0.9945	0.8081, 1.1810	< 0.0001				
NLR	(continuous)	2167	−1.1193	−1.4593, −0.7793	< 0.0001				

*Note:* The variables were based on baseline data (at the index date or within 1 year before the index date).

Abbreviations: BMI, body mass index; CCI, Charlson comorbidity index; CI, confidence interval; CONUT, controlling nutritional status; CP, chronic pancreatitis; CRP, C‐reactive protein; mGPS, modified Glasgow prognostic score; NLR, neutrophil‐to‐lymphocyte ratio; PEI, pancreatic exocrine insufficiency; PFD, pancreatic function diagnostic test.

## 4 Discussion

In this database study using medical claims data from April 2008 to August 2022, we observed an increase in the number of patients diagnosed with CP, prescribed PERT, and diagnosed with PEI over time in Japan. Patients in the PERT cohort were more likely to be underweight and have comorbidities, such as PEI, diabetes mellitus, and abdominal pain, than those in the no‐PERT cohort, suggesting that patients with CP in the advanced decompensated stage were more likely to be prescribed PERT. Most patients with CP started PERT within 1 year of the diagnosis; however, the median PERT persistence time was short (254.0 days), with a 5‐year persistence rate of 20.4%. Although the median event‐free survival was shorter in the PERT cohort than in the no‐PERT cohort, this may reflect the more severe disease burden and comorbidity profile of patients receiving PERT rather than the effect of PERT itself.

In this study, more patients in the PERT cohort showed signs of malnutrition (e.g., BMI < 18.5 kg/m^2^, a higher CONUT score, and a cachexia category in the mGPS) and gastrointestinal symptoms (abdominal pain and distention) than those in the no‐PERT cohort. The proportions of patients with alcoholic chronic pancreatitis and those who smoked—an established risk factor for acute and chronic pancreatitis [19]—were also higher in the PERT cohort. Although a previous study found that fecal fat excretion correlated with pancreatic exocrine dysfunction [20], the proportion of patients who underwent steatorrhea‐related tests and were diagnosed with steatorrhea was low in both cohorts, suggesting that steatorrhea is not routinely assessed in patients with CP in Japan.

After the Japanese clinical diagnostic criteria for CP were revised in 2019 [16], the proportion of patients who started PERT within 1 year of CP diagnosis remained high at 86.7% in Term 2 and 79.8% in Term 3; however, the proportion of patients with a PEI diagnosis in the PERT cohort increased from 47.3% in Term 2 to 54.5% in Term 3. This increase may be due to a larger number of patients undergoing PEI screening tests or due to clinical PEI diagnosis, given that 61.1% of patients started PERT within 1 month of CP diagnosis.

The approved dose of pancrelipase for treating poor digestion in patients with PEI is 600 mg per meal, three times a day (1800 mg/day), which can be adjusted according to the patient's condition [14]. In this study, the 900 and 1800 mg doses accounted for approximately 40% of each dose. The reason for the reduced daily dose of 900 mg is unclear, but it might be to improve treatment adherence and balance cost and benefit.

We found that the number of patients with PERT prescriptions decreased with every prescription, and the median PERT persistence time was shorter than a year (254.0 days). The persistence rate, estimated from prescriptions made at doctor visits in this study, also decreased from 44.3% at year 1 to 28.1% at year 3 and 20.4% at year 5. Although the median persistence time was longer in patients with PEI diagnoses, PFD, or nutritional guidance, further research is needed to understand the factors that facilitate regular doctor visits among patients with CP.

Multivariable analysis revealed that the risk of CP‐related outcome events increased in older patients, those with alcoholic CP, and those with comorbidities (higher CCI). Compared with patients in the no‐PERT cohort, those in the PERT cohort experienced more in‐hospital deaths (6.3% vs. 4.7%) and weight loss (13.3% vs. 9.2%), and had a shorter median event‐free survival time (2386.0 vs. 3442.0 days). Although no causal relationships between PERT and clinical outcomes could be established in this observational study, we believe this does not indicate that PERT changed the prognosis of patients, but rather that PERT was more likely to be prescribed to patients whose prognosis was poor at baseline, such as those in the advanced decompensated stage or those with malignancy (including pancreatic cancer).

Despite the strength of analyzing a large‐scale claims database in Japan, this study has some limitations. First, this observational study was designed to descriptively characterize patients with CP receiving PERT in real‐world clinical practice, rather than to estimate the effects of PERT on clinical outcomes. Because propensity score matching or other approaches to adjust for confounding were not applied, the observed difference between the PERT and no‐PERT cohorts may reflect differences in baseline patient characteristics and other confounding factors and should not be interpreted as a causal effect of PERT. Second, detailed clinical data of the patients, such as the reason for PERT discontinuation or death outside the hospital, were not available in the MDV database. Third, because the MDV database collects data from advanced care hospitals using the DPC system, this study did not include data on patients treated at other institutions. Caution is necessary when generalizing the findings of this study to a broader population. Fourth, PEI was identified by specialists based on disease names and ICD‐10 codes rather than on pancreatic function or steatorrhea‐related test results, which may have led to missed or incorrect diagnoses.

In summary, we observed an increase in the number of PEI diagnoses and PERT prescriptions among patients with CP in Japan over the past decade. Most patients with CP started PERT within 1 year of the diagnosis, but the long‐term persistence rate was low. The low PERT persistence rate observed in this study suggests that further research is needed to understand the factors that affect treatment persistence among patients with CP.

## Funding

Viatris Pharmaceuticals Japan G.K. funded this study.

## Ethics Statement

Based on the applicable legal requirements in Japan, approval from the institutional review board and informed consent from patients were not required for this study, as it used anonymized data.

## Consent

The authors have nothing to report.

## Conflicts of Interest

Noriharu Itoh, Yuko Hasebe, and Kazutaka Nozawa are Viatris Pharmaceuticals Japan G.K. employees. Toru Yada was an employee of EPS Corporation when the study was conducted and is currently employed by Seven to One Inc.

## Supporting information


**Figure S1:** Study design for the prevalent CP cohort (A), incident CP cohort (B), and PERT cohort (C). CP, chronic pancreatitis; PERT, pancreatic enzyme replacement therapy.
**Table S1:** PERT prescriptions in the prevalent and incident CP cohorts.
**Table S2:** Time from the first date of CP diagnosis to the start date of PERT in the PERT cohort.

## Data Availability

The data that support the findings of this study were obtained from the MDV claims database and are not publicly available due to contractual and ethical restrictions. Researchers who meet the criteria for access to this data may obtain the data from Medical Data Vision Co. Ltd. under a data use agreement and in compliance with applicable laws and regulations.
