# Supplementary material for: Current Status of Pancreatic Exocrine Insufficiency Diagnosis and Pancreatic Enzyme Replacement Therapy Implementation in Patients With Chronic Pancreatitis
Source: JGH Open. 2026 Sep 29;10(10):e70473. doi: 10.1002/jgh3.70473 (PMC13624702; doi:10.1002/jgh3.70473)
Supplement: Supplementary file 1 [file JGH3-10-e70473-s001.docx]

**Current Status of Pancreatic Exocrine Insufficiency Diagnosis and Pancreatic Enzyme Replacement Therapy Implementation in Patients with Chronic Pancreatitis**

Noriharu Itoh et al.

**Fig. S1.** Study design for the prevalent CP cohort (A), incident CP cohort (B), and PERT cohort (C). CP, chronic pancreatitis; PERT, pancreatic enzyme replacement therapy.

**Table S1.** PERT prescriptions in the prevalent and incident CP cohorts

|  | Prevalent CP cohort | | | Incident CP cohort | | |
| --- | --- | --- | --- | --- | --- | --- |
|  | Overall | Term 2 | Term 3 | Overall | Term 2 | Term 3 |
|  | N = 103,064 | N = 67,120 | N = 64,104 | N = 35,925 | N = 19,244 | N = 16,168 |
| PERT prescriptions, yes, n (%) | 9,767 (9.5) | 5,450 (8.1) | 7,878 (12.3) | 2,985 (8.3) | 1,585 (8.2) | 1,391 (8.6) |
| Initial prescription dose, mg, mean (SD) | 1,263.0 (783.5) | 1,273.5 (934.9) | 1,257.2 (784.8) | 1,256.8 (767.5) | 1,255.7 (683.9) | 1,258.0 (854.5) |
| Duration of prescriptions, days, mean (SD) | 602.9 (796.8) | 473.0 (576.9) | 719.7 (853.9) | 445.0 (638.6) | 603.4 (779.7) | 254.6 (304.2) |

The inclusion period (April 2009 to August 2022) was divided into the following terms: Term 1, April 2009 to July 2011 (before the launch of pancrelipase); Term 2, August 2011 to December 2018 (after the launch of pancrelipase, before the CP diagnostic criteria changed in 2019); and Term 3, January 2019 to August 2022 (after the CP diagnostic criteria changed in 2019).

CP, chronic pancreatitis; PERT, pancreatic enzyme replacement therapy; SD, standard deviation.

**Table S2.** Time from the first date of CP diagnosis to the start date of PERT in the PERT cohort

| Time from the first date of CP diagnosis to the PERT start date | Overall period | Term 2 | Term 3 |
| --- | --- | --- | --- |
|  | N = 3,056 | N = 1,396 | N = 1,660 |
| ≤1 month | 1,867 (61.1) | 843 (60.4) | 1,024 (61.7) |
| >1 and ≤3 months | 326 (10.7) | 184 (13.2) | 142 (8.6) |
| >3 and ≤6 months | 153 (5.0) | 75 (5.4) | 78 (4.7) |
| >6 month and ≤1 year | 188 (6.2) | 108 (7.7) | 80 (4.8) |
| ≤1 year | 2,534 (82.9) | 1,210 (86.7) | 1,324 (79.8) |
| >1 and ≤3 years | 323 (10.6) | 135 (9.7) | 188 (11.3) |
| >3 and ≤5 years | 125 (4.1) | 41 (2.9) | 84 (5.1) |
| >5 years | 74 (2.4) | 10 (0.7) | 64 (3.9) |

Term 2 was between August 2011 and December 2018 (after the launch of pancrelipase, before the CP diagnostic criteria were changed in 2019), and Term 3 was between January 2019 and August 2022 (after the CP diagnostic criteria were changed in 2019). CP, chronic pancreatitis; PERT, pancreatic enzyme replacement therapy.
